# Author Correction: HIF1α inhibition facilitates Leflunomide-AHR-CRP signaling to attenuate bone erosion in CRP-aberrant rheumatoid arthritis

**DOI:** 10.1038/s41467-020-16901-6

**Published:** 2020-06-22

**Authors:** Chao Liang, Jie Li, Cheng Lu, Duoli Xie, Jin Liu, Chuanxin Zhong, Xiaohao Wu, Rongchen Dai, Huarui Zhang, Daogang Guan, Baosheng Guo, Bing He, Fangfei Li, Xiaojuan He, Wandong Zhang, Bao-Ting Zhang, Ge Zhang, Aiping Lu

**Affiliations:** 1 Law Sau Fai Institute for Advancing Translational Medicine in Bone and Joint Diseases, School of Chinese Medicine, Hong Kong Baptist University, 999077 Hong Kong SAR, China; 2 Institute of Integrated Bioinfomedicine and Translational Science, School of Chinese Medicine, Hong Kong Baptist University, 999077 Hong Kong SAR, China; 3 Institute of Precision Medicine and Innovative Drug Discovery, HKBU Institute for Research and Continuing Education, 518000 Shenzhen, China; 4 Faculty of Medicine, School of Chinese Medicine, Chinese University of Hong Kong, 999077 Hong Kong SAR, China; 5 Institute of Basic Research in Clinical Medicine, China Academy of Chinese Medical Sciences, 100700 Beijing, China; 6 The First Affiliated Hospital of Anhui University of Chinese Medicine, 230031 Hefei, China; 7 Institute of Arthritis Research, Shanghai Academy of Chinese Medical Sciences, Guanghua Integrative Medicine Hospital/Shanghai University of TCM, 200032 Shanghai, China

**Keywords:** Bone, Rheumatoid arthritis

Correction to: *Nature Communications* 10.1038/s41467-019-12163-z, published online 8 October 2019.

This Article contained errors in Figs. 4i and 5a, for which the authors apologise. The lanes labelled ARNT (lower row) in Fig. 4i and HIF1alpha in Fig. 5a were not from the membranes used to probe for the other proteins shown in each panel. These have now been replaced with the correct rows from the same membranes. These errors were reproduced in the source data file that contains the uncropped membranes. The errors did not affect the conclusions of the manuscript. They have now been corrected both the PDF and HTML versions of the Article, and the Source Data file has been amended to include the correct images of the respective uncropped membranes.

