# Supplementary material for: HIF1α inhibition facilitates Leflunomide-AHR-CRP signaling to attenuate bone erosion in CRP-aberrant rheumatoid arthritis
Source: Nat Commun. 2019 Oct 8;10:4579. doi: 10.1038/s41467-019-12163-z (PMC6783548; doi:10.1038/s41467-019-12163-z)
Supplement: Supplementary file 1 — Supplementary Information [file 41467_2019_12163_MOESM1_ESM.pdf]

Supplementary Information

HIF1α inhibition facilitates Leflunomide-AHR-CRP signaling to attenuate bone erosion in CRP-aberrant rheumatoid arthritis

Liang C. *et. al.*

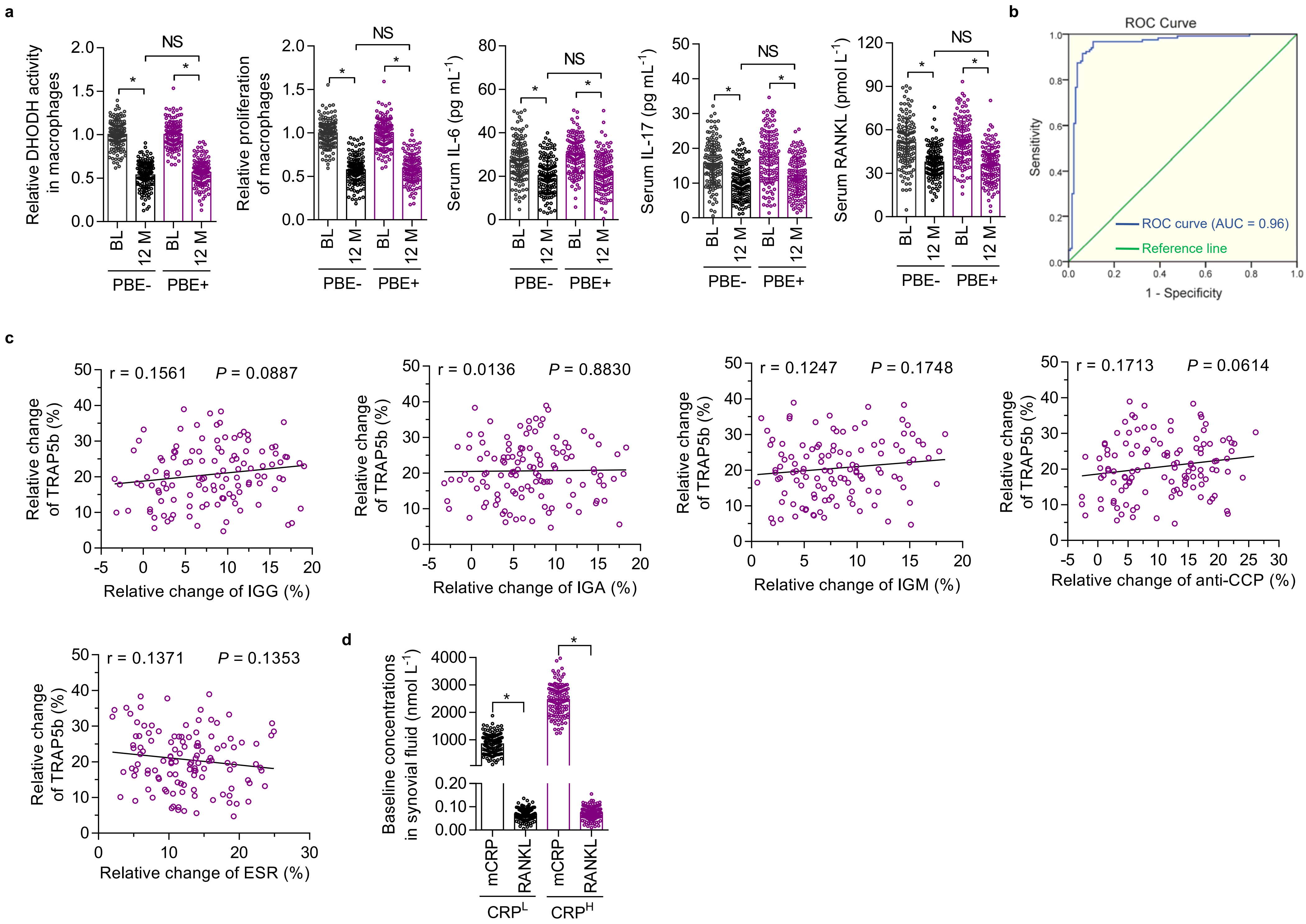
a
Relative DHODH activity in macrophages
Relative proliferation of macrophages
Serum IL-6 (pg mL-1)
Serum IL-17 (pg mL-1)
Serum RANKL (pmol L-1)
NS
*
BL
12 M
PBE-
PBE+
b
ROC Curve
Sensitivity
1 - Specificity
ROC curve (AUC = 0.96)
Reference line
c
r = 0.1561 P = 0.0887
r = 0.0136 P = 0.8830
r = 0.1247 P = 0.1748
r = 0.1713 P = 0.0614
r = 0.1371 P = 0.1353
Relative change of TRAP5b (%)
Relative change of IGG (%)
Relative change of IGA (%)
Relative change of IGM (%)
Relative change of anti-CCP (%)
Relative change of ESR (%)
d
Baseline concentrations in synovial fluid (nmol L-1)
mCRP
RANKL
CRPL
CRPH

Supplementary Fig. 1. Effects of Leflunomide on macrophages and cytokines, ROC and correlation analysis. (**a**) Relative levels of DHODH activity and proliferation of macrophages in synovial fluid and IL-6, IL-17 and RANKL levels in serum from progressive bone erosion-positive (PBE+, n = 120) and progressive bone erosion-negative (PBE-, n = 130) RA patients before (BL) and after (12 M) treatment. The level of DHODH activity and macrophage proliferation were normalized to the corresponding baseline (BL) in PBE+ (n = 120) and PBE- (n = 130) RA patients. * $P < 0.05$ as determined by repeated-measures analysis of variance (ANOVA) with a *post-hoc* test, NS: no significance. (**b**) Receiver operating characteristic (ROC) analysis of baseline CRP with progressive bone erosion in RA patients**.** The area under the curve (AUC) was 0.96. (**c**) Correlation between the relative change of TRAP5b with blood indicators from the corresponding baseline in CRP^H RA patients (n = 120) as determined by Pearson's correlation. (**d**) Baseline concentrations of monomeric CRP (mCRP) and receptor activator of nuclear factor kappa-B ligand (RANKL) in synovial fluid from PBE- (n = 130) and PBE+ (n = 120) RA patients. * $P < 0.05$ as determined by two-sided *t*-test. IGM: Immunoglobulin M, IGG: Immunoglobulin G, IGA: Immunoglobulin A, anti-CCP: anti-cyclic citrullinated peptide antibody, ESR: erythrocyte sedimentation rate, TRAP5b: tartrate-resistant acid phosphatase 5b. Source data are provided as a Source Data file.

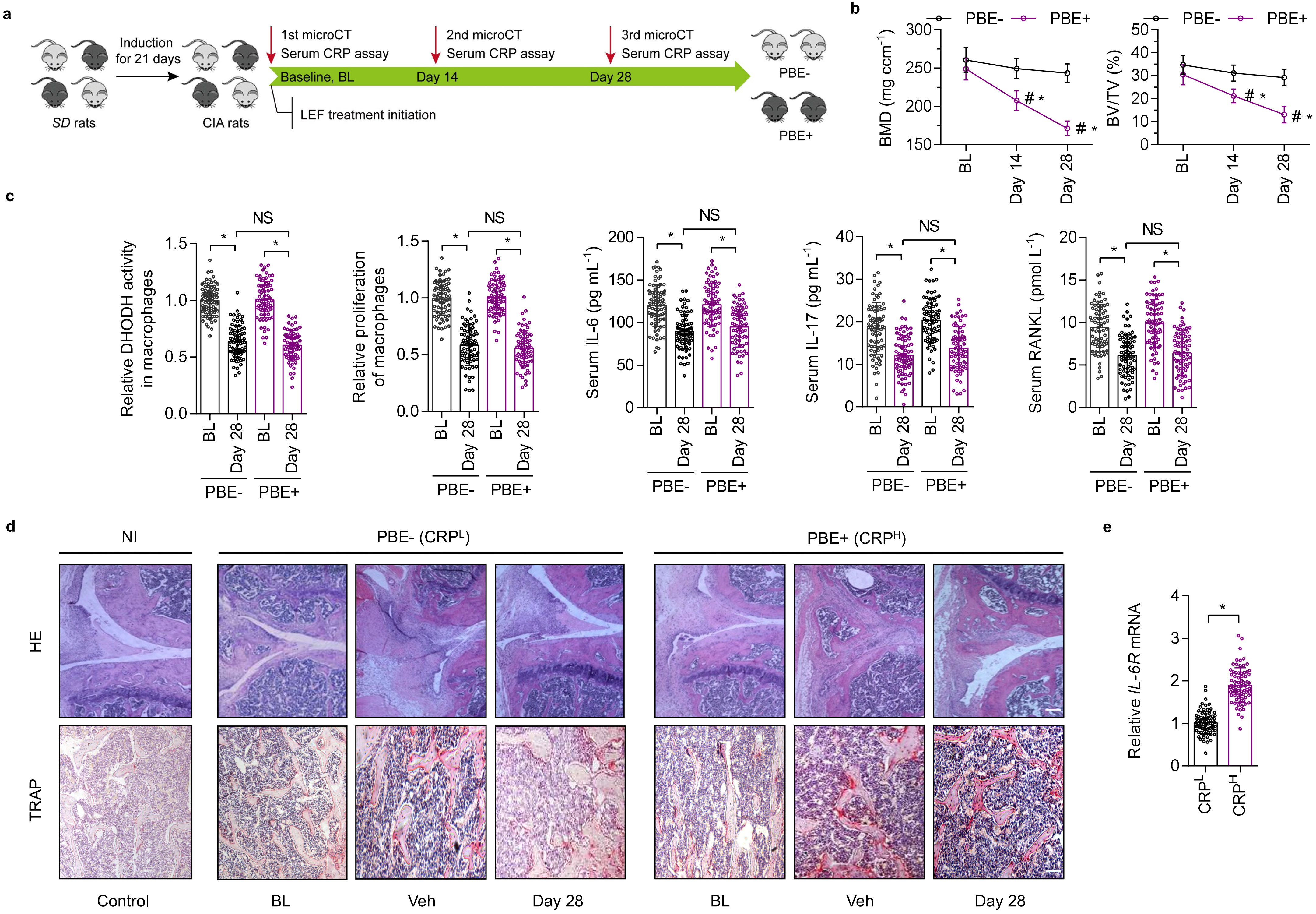

a
Induction for 21 days
SD rats
CIA rats
1st microCT
Serum CRP assay
2nd microCT
Serum CRP assay
3rd microCT
Serum CRP assay
Baseline, BL
Day 14
Day 28
LEF treatment initiation
PBE-
PBE+
b
PBE-
PBE+
BMD (mg ccm-1)
BV/TV (%)
BL
Day 14
Day 28
c
Relative DHODH activity in macrophages
Relative proliferation of macrophages
Serum IL-6 (pg mL-1)
Serum IL-17 (pg mL-1)
Serum RANKL (pmol L-1)
NS
d
NI
PBE- (CRPL)
PBE+ (CRPH)
HE
TRAP
Control
BL
Veh
Day 28
e
Relative IL-6R mRNA
CRPL
CRPH

Supplementary Fig. 2. Immune response, bone mass and osteoclastic activity in PBE+ and PBE- CIA rats. (**a**) The diagram of the experimental design. Briefly, *Sprague-Dawley* (*SD*) rats were immunized with bovine type II collagen for 21 days to establish the collagen-induced-arthritis (CIA) model. The CIA rats were orally administered with Leflunomide for 28 days at a dose of 10 mg kg^{-1} d^{-1}. *In vivo* microCT analysis and serum CRP assay was performed before (baseline, BL) and after the treatment at day 14 and day 28. (**b**) Bone mass parameters (BMD and BV/TV) in PBE- rats (n = 77) and PBE+ rats (n = 73) before (BL) and after the treatment. * $P < 0.05$ for day 14 or day 28 versus BL in PBE+ and # $P < 0.05$ for PBE+ versus PBE- as determined by repeated measures ANOVA with a *post-hoc* test. (**c**) Relative level of DHODH activity and proliferation of macrophages in synovial fluid and IL-6, IL-17 and RANKL levels in serum from PBE+ rats (n = 73) and PBE- rats (n = 77). The level of DHODH activity and proliferation of macrophages were normalized to the corresponding baseline in PBE+ and PBE- CIA rats before treatment. * $P < 0.05$ as determined by one-way ANOVA with a *post-hoc* test, NS: no significance. (**d**) Histological analysis by hematoxylin and eosin (H&E) and TRAP staining to document inflammatory synovial hyperplasia and osteoclastic activity in PBE+ (CRP^{H}) and PBE- (CRP^{L}) CIA rats. Scale bar, 40 μm. (**e**) The relative baseline *IL-6R* mRNA level in livers from CRP^{L} (n = 77) and CRP^{H} CIA rats (n = 73). * $P < 0.05$ as determined by two-sided *t*-test. Source data are provided as a Source Data file.

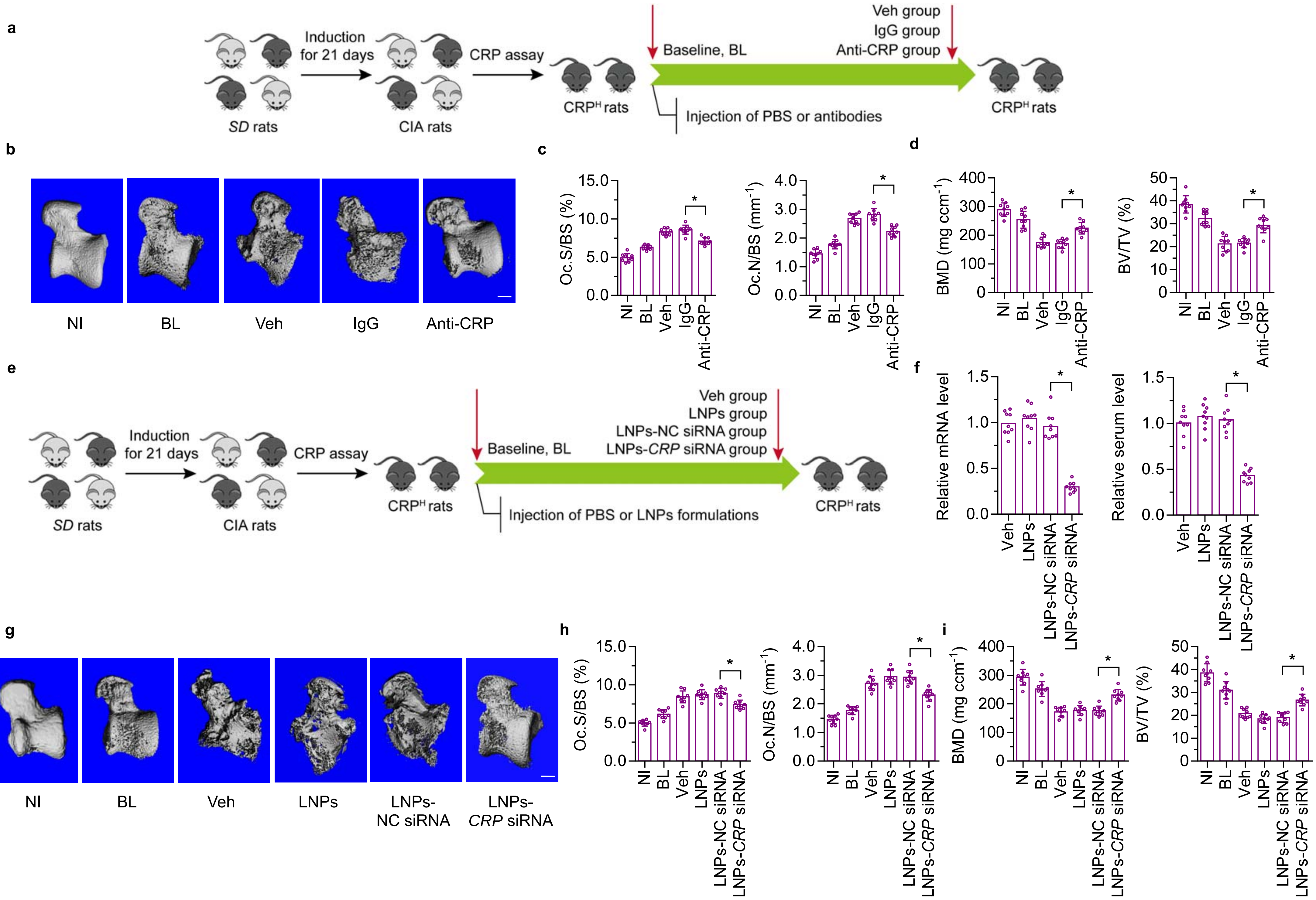

a
SD rats
Induction for 21 days
CIA rats
CRP assay
CRP^H rats
Baseline, BL
Veh group
IgG group
Anti-CRP group
Injection of PBS or antibodies
b
NI
BL
Veh
IgG
Anti-CRP
c
Oc.S/BS (%)
Oc.N/BS (mm-1)
d
BMD (mg ccm-1)
BV/TV (%)
e
LNPs group
LNPs-NC siRNA group
LNPs-CRP siRNA group
Injection of PBS or LNPs formulations
f
Relative mRNA level
Relative serum level
LNPs
LNPs-NC siRNA
LNPs-CRP siRNA
g
h
i

Supplementary Fig. 3. Effects of CRP inhibition on bone erosion in CRP^H CIA rats. (**a**) The diagram of the experimental design using the anti-CRP antibody. Briefly, *SD* rats were immunized with bovine type II collagen for 21 days to establish the CIA model. CIA rats with CRP level within the established reference range for CRP^H rats (91.24 ± 10.12 mg L^{-1}) were intra-articularly injected with PBS (vehicle, Veh), rabbit IgG controls (IgG, 20 μg for each rat) or rabbit anti-rat CRP antibodies (Anti-CRP, 20 μg for each rat) at ankle joints every week for 28 days. (**b**) The representative reconstructed three-dimensional micro-CT images of the talus bone from the CRP^H rats before (baseline, BL) and after treatment. Non-immunized (NI) rats were used as controls of CIA rats. Scale bar, 1.0 mm. (**c**) Bone resorption parameters including osteoclast surface per bone surface (Oc.S/BS) and osteoclast number per bone surface (Oc.N/BS) of the talus bone. (**d**) Bone mass parameters including bone mineral density (BMD) and bone volume per total volume (BV/TV) of the talus bone. (**e**) The diagram of the experimental design using the *CRP* siRNA. Briefly, the CIA rats with CRP^H were intravenously administrated with PBS (Veh), LNPs, LNPs-negative control siRNA (LNPs-NC siRNA) or LNPs-*CRP* siRNA every two weeks for 28 days, with a dosage of siRNA at 5.0 mg kg^{-1}. (**f**) Relative levels of *CRP* mRNA in hepatocytes and CRP release in serum from CRP^H rats. (**g**) The representative reconstructed three-dimensional micro-CT images of the talus bone. Scale bar, 1.0 mm. (**h**) Bone resorption parameters including Oc.S/BS and Oc.N/BS of the talus bone. (**i**) Bone mass parameters including BMD and BV/TV of the talus bone. * $P < 0.05$ as determined by one-way ANOVA with a *post-hoc* test. n = 9 for each group. Source data are provided as a Source Data file.

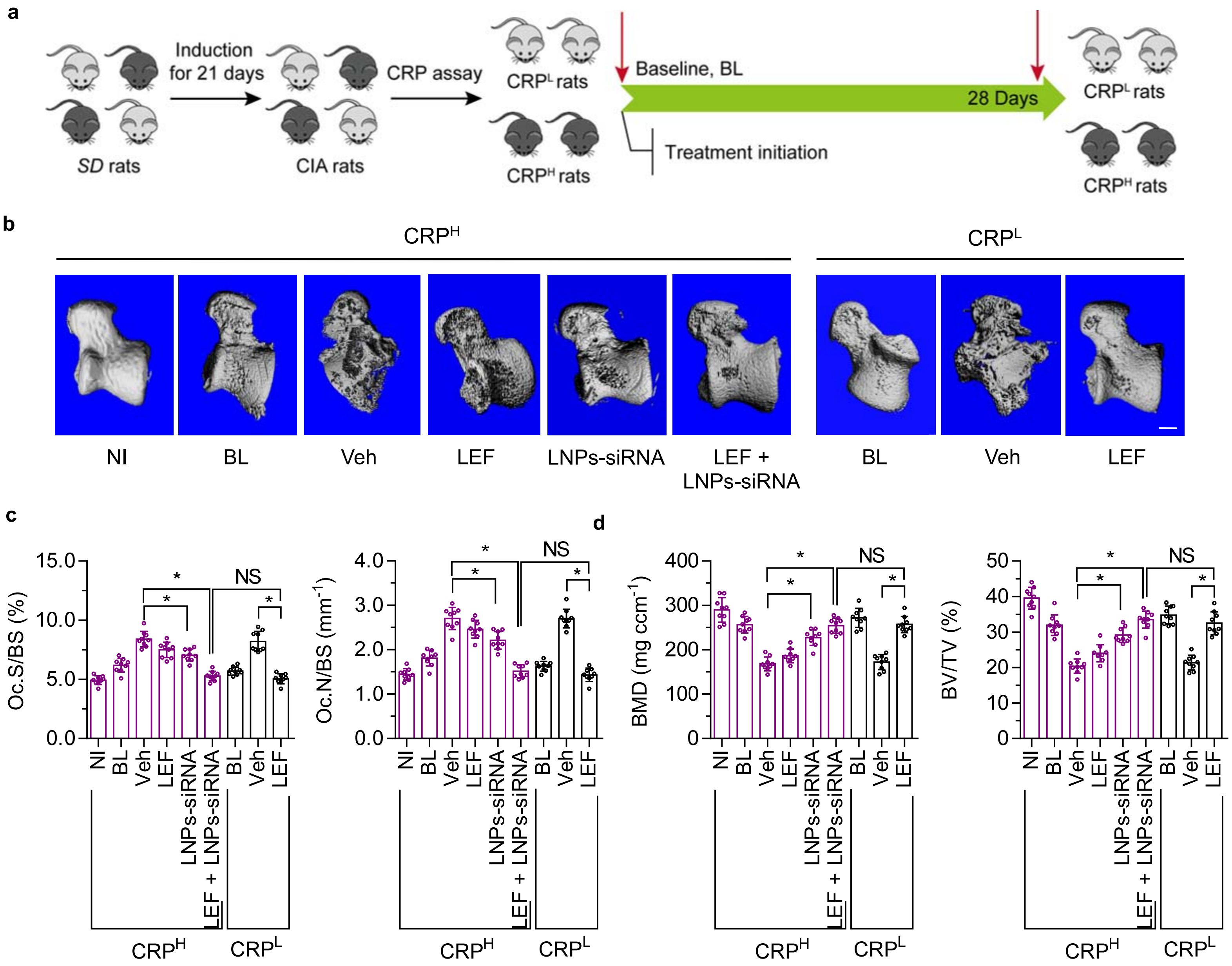
a
Induction for 21 days
SD rats
CIA rats
CRP assay
CRP^L rats
CRP^H rats
Baseline, BL
28 Days
Treatment initiation
b
CRP^H
CRP^L
NI
BL
Veh
LEF
LNPs-siRNA
LEF + LNPs-siRNA
c
d
Oc.S/BS (%)
Oc.N/BS (mm^-1)
BMD (mg ccm^-1)
BV/TV (%)
NS
*

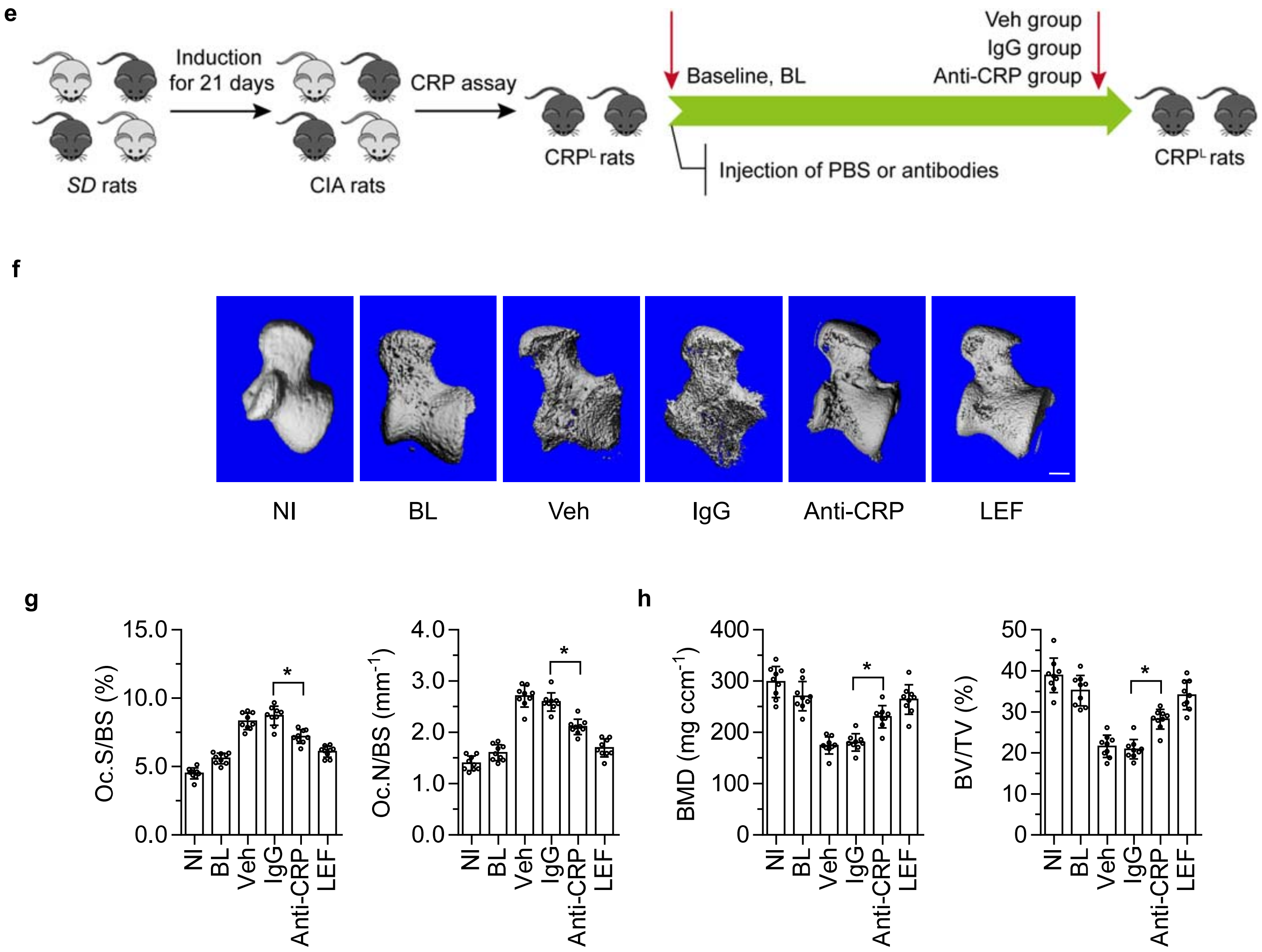
e
Induction for 21 days
CRP assay
Baseline, BL
Veh group
IgG group
Anti-CRP group
SD rats
CIA rats
CRP^L rats
Injection of PBS or antibodies
CRP^L rats
f
NI
BL
Veh
IgG
Anti-CRP
LEF
g
Oc.S/BS (%)
Oc.N/BS (mm^{-1})
h
BMD (mg ccm^{-1})
BV/TV (%)
NI
BL
Veh
IgG
Anti-CRP
LEF

Supplementary Fig. 4. Effects of CRP inhibition on bone erosion in CIA rats. (**a**) The diagram of the experimental design. Briefly, *SD* rats were immunized with bovine type II collagen for 21 days to establish the CIA models. The CIA rats were classified into CRP^H and CRP^L subgroups based on their established reference range of CRP. The CRP^H CIA rats were treated with vehicle (Veh), Leflunomide (LEF, 10 mg kg^{-1} d^{-1}), LNPs-CRP siRNA or Leflunomide in combination with LNPs-CRP siRNA for 28 days. The CRP^L CIA rats were treated with vehicle (Veh) or Leflunomide (LEF, 10 mg kg^{-1} d^{-1}) alone for 28 days. (**b**) The representative reconstructed three-dimensional micro-CT images of the talus bone from the CRP^H and CRP^L CIA rats before (baseline, BL) and after treatment. Scale bar, 1.0 mm. (**c**) Bone resorption parameters including Oc.S/BS and Oc.N/BS of the talus bone. (**d**) Bone mass parameters including BMD and BV/TV of the talus bone. (**e**) The diagram of the experimental design. Briefly, *SD* rats were immunized with bovine type II collagen for 21 days to establish the CIA model. The CIA rats with CRP level within the established reference range for CRP^L rats (52.45 ± 7.54 mg L^{-1}) were intra-articularly injected with PBS (vehicle, Veh), rabbit IgG controls (IgG, 20 μg for each rat) or anti-CRP antibodies (Anti-CRP, 20 μg for each rat) at ankle joints every week for 28 days. (**f**) The representative reconstructed three-dimensional micro-CT images of the talus bone from the CRP^L rats before (baseline, BL) and after treatment. Scale bar, 1.0 mm. (**g**) Bone resorption parameters including Oc.S/BS and Oc.N/BS of the talus bone. (**h**) Bone mass parameters including BMD and BV/TV of the talus bone. * $P < 0.05$ as determined by one-way ANOVA with a *post-hoc* test. n = 9 for each group. Source data are provided as a Source Data file.

a

b

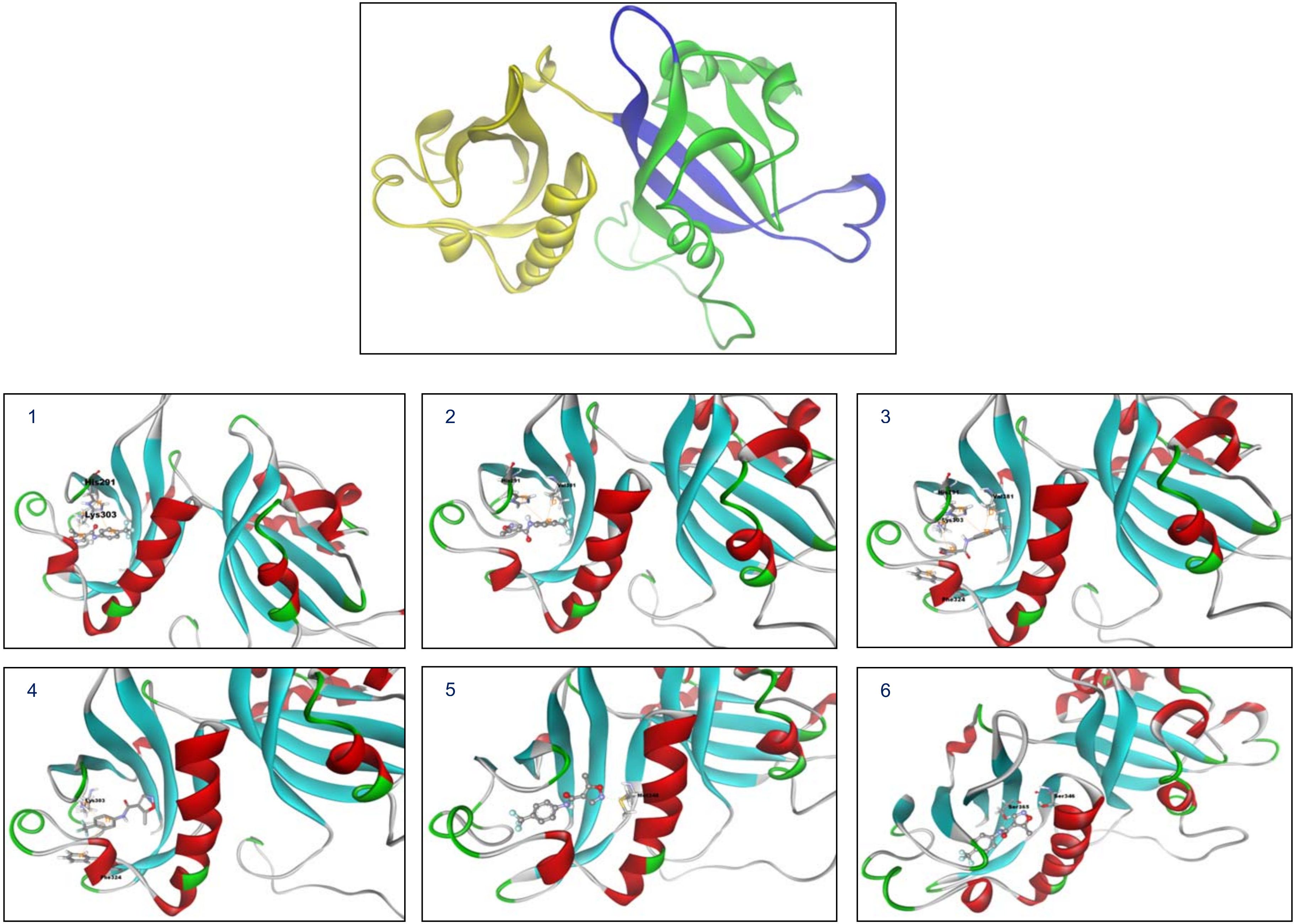

Supplementary Fig. 5. Molecular docking between AHR PAS-A and PAS-B domains with Leflunomide. (**a**) Homology modeling of AHR PAS-A and PAS-B domains. After loop refinement and energy minimization, the optimal structure was generated by MODELER. PAS-A domain was shown in green color and PAS-B domain was shown in yellow color. The beta-sheet that connected PAS-A and PAS-B domains was shown in blue color. (**b**) Six top-ranked binding modes between AHR PAS-A and PAS-B domains and Leflunomide with low energies generated by AutoDock Vina.

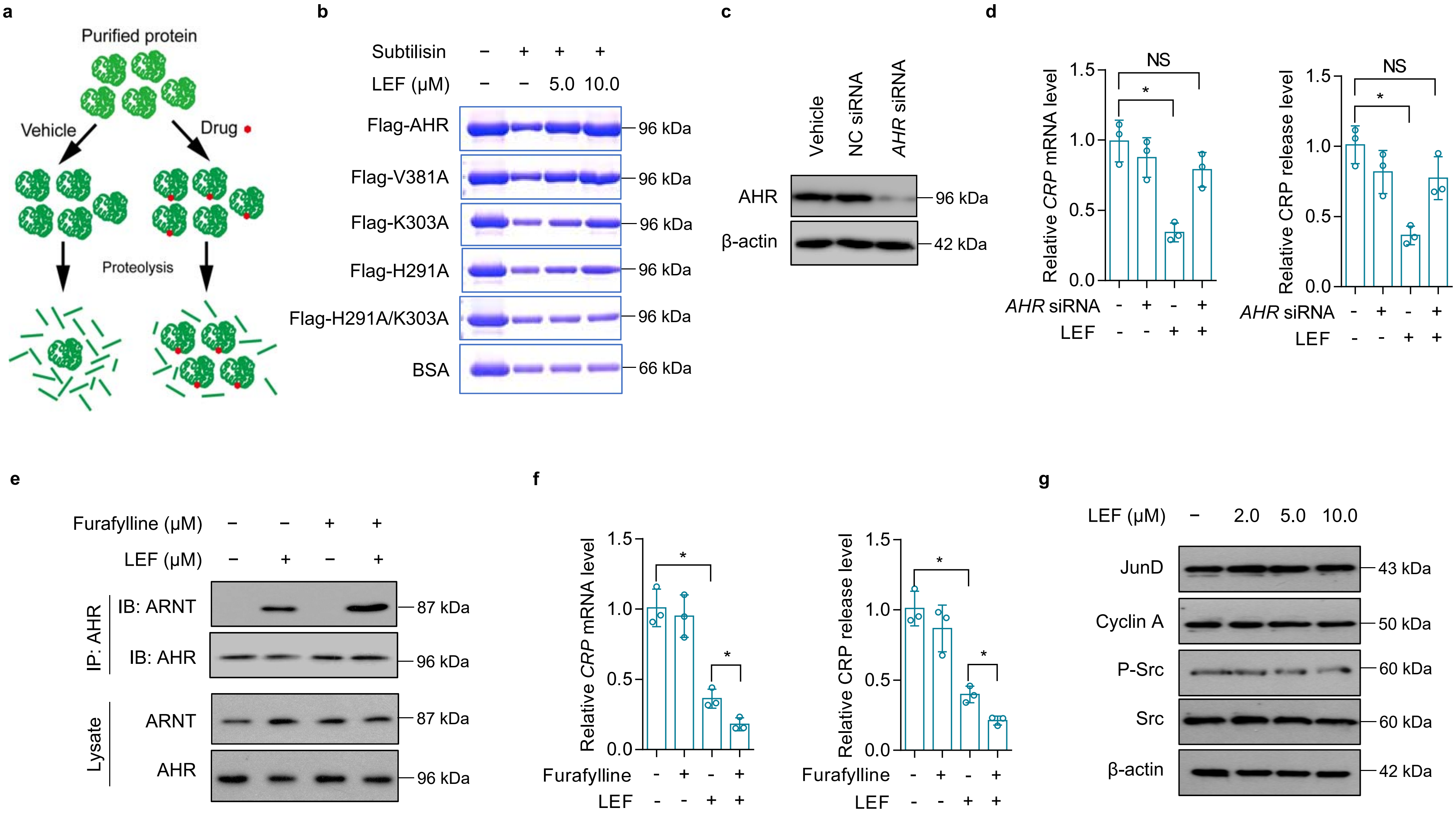

a
Purified protein
Vehicle
Drug
Proteolysis
b
Subtilisin
LEF (μM)
5.0
10.0
Flag-AHR
Flag-V381A
Flag-K303A
Flag-H291A
Flag-H291A/K303A
BSA
96 kDa
66 kDa
c
Vehicle
NC siRNA
AHR siRNA
AHR
β-actin
96 kDa
42 kDa
d
Relative CRP mRNA level
Relative CRP release level
NS
AHR siRNA
LEF
e
Furafylline (μM)
LEF (μM)
IP: AHR
IB: ARNT
IB: AHR
Lysate
ARNT
AHR
87 kDa
96 kDa
f
Relative CRP mRNA level
Relative CRP release level
Furafylline
LEF
g
LEF (μM)
2.0
5.0
10.0
JunD
Cyclin A
P-Src
Src
β-actin
43 kDa
50 kDa
60 kDa
42 kDa

Supplementary Fig. 6. DARTS assay and effects of Leflunomide on AHR genomic and non-genomic pathways. (**a**) The diagram of the experimental design. 30.0 μg Flag-AHR, Flag-AHR mutants (H291A, K303A and V381A) or BSA were incubated with Leflunomide (5.0 and 10.0 μM) or DMSO (vehicle), followed by digestion with proteases (subtilisin). (**b**) Protein bands protected from proteolysis conferred by the interaction with Leflunomide (LEF). After incubation with Leflunomide or DMSO in the presence of subtilisin, the samples were separated by sodium dodecyl sulfate polyacrylamide gel electrophoresis (SDS-PAGE) and stained by coomassie brilliant blue. (**c**) Level of AHR expression in human normal hepatocytes (THLE-2 cells) transfected with vehicle, *AHR* siRNA and negative control siRNA (NC siRNA), respectively. (**d**) Relative *CRP* mRNA (left) and CRP release (right) levels in THLE-2 cells transfected with *AHR* siRNA in presence of Leflunomide (LEF). (**e**) Binding of ARNT with AHR (AHR activation) determined by immunoprecipitation in human normal primary hepatocytes incubated with vehicle (DMSO) and Leflunomide (LEF, 10.0 μM) in presence of Furafylline (20.0 μM) or not, respectively. (**f**) Relative levels of *CRP* mRNA expression (left) and CRP release (right) in human normal primary hepatocytes with the above treatments. (**g**) Expression of JunD and cyclin A and Src activation (phosphorylation of Src, p-Src) in THLE-2 cells incubated with vehicle (DMSO) and Leflunomide at a series of concentrations, respectively. * $P < 0.05$ as determined by one-way ANOVA with a *post-hoc* test. Each experiment was repeated three times. Source data are provided as a Source Data file.

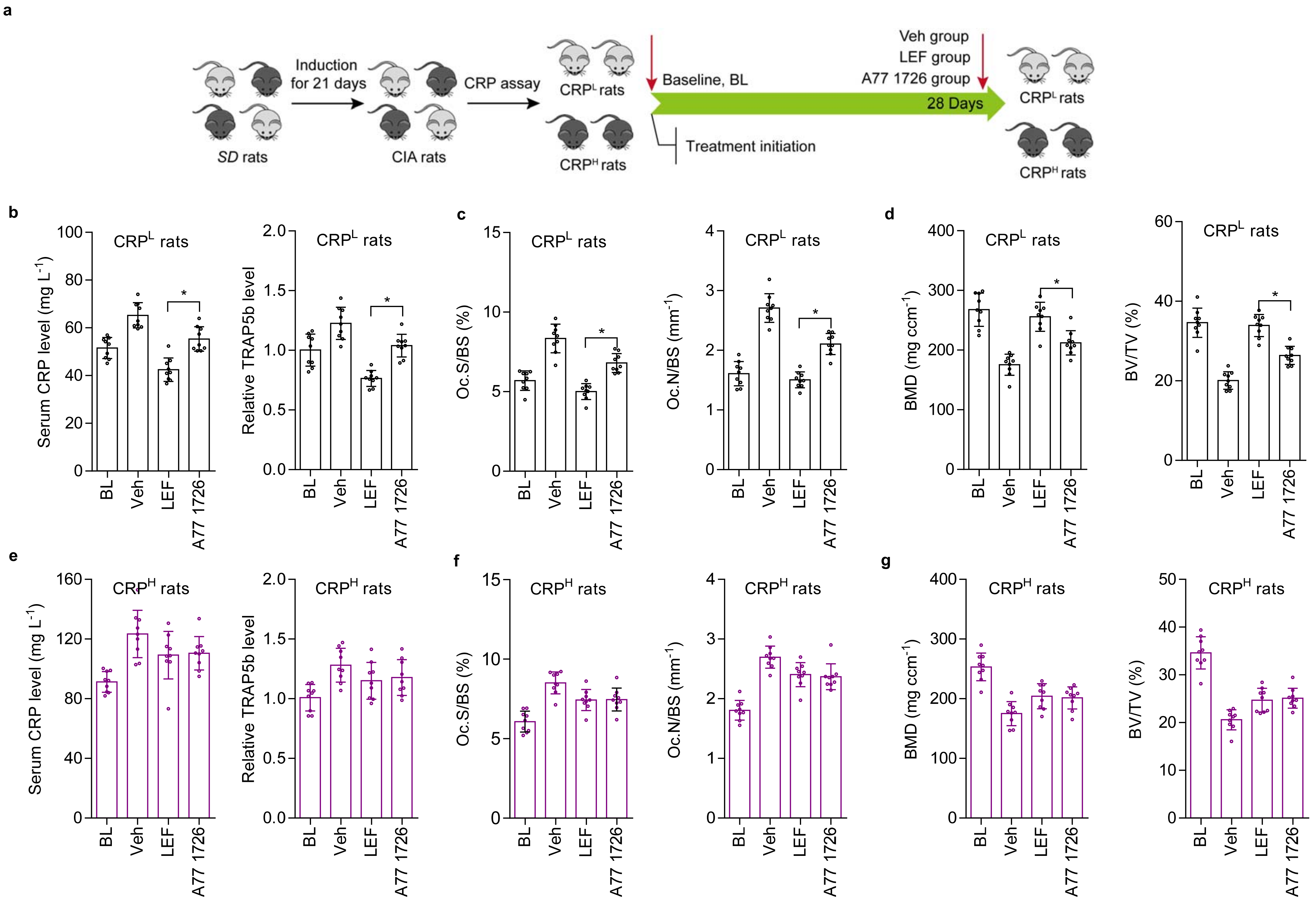

a
Induction for 21 days
CRP assay
SD rats
CIA rats
CRP^L rats
CRP^H rats
Baseline, BL
Veh group
LEF group
A77 1726 group
28 Days
Treatment initiation
b
c
d
e
f
g
CRP^L rats
CRP^H rats
Serum CRP level (mg L^-1)
Relative TRAP5b level
Oc.S/BS (%)
Oc.N/BS (mm^-1)
BMD (mg ccm^-1)
BV/TV (%)
BL
Veh
LEF
A77 1726

Supplementary Fig. 7. Effects of Leflunomide and A77 1726 in CRP^L or CRP^H CIA rats. (**a**) The diagram of the experimental design. Briefly, *SD* rats were immunized with bovine type II collagen for 21 days to establish the CIA models. The CIA rats were classified into CRP^H and CRP^L subgroups based on their established reference range of CRP. The CRP^H and CRP^L rats were orally administered with vehicle (Veh), Leflunomide (LEF) and A77 1726 for 28 days at a dose of 10 mg kg^{-1} d^{-1}, respectively. (**b**) Levels of serum CRP and TRAP5b in CRP^L rats before (baseline, BL) and after treatment with vehicle (Veh), Leflunomide (LEF) and A77 1726, respectively. (**c**) Bone resorption parameters including Oc.S/BS and Oc.N/BS in CRP^L rats. (**d**) Bone mass parameters including BMD and BV/TV in CRP^L rats. (**e**) Levels of serum CRP and TRAP5b in CRP^H rats before (BL) and after treatment with vehicle (Veh), Leflunomide (LEF) and A77 1726, respectively. (**f**) Bone resorption parameters including Oc.S/BS and Oc.N/BS determined by bone histomorphometric analysis in CRP^H rats. (**g**) Bone mass parameters including BMD and BV/TV in CRP^H rats. * $P < 0.05$ as determined by one-way ANOVA with a *post-hoc* test. n = 9 for each group. Source data are provided as a Source Data file.

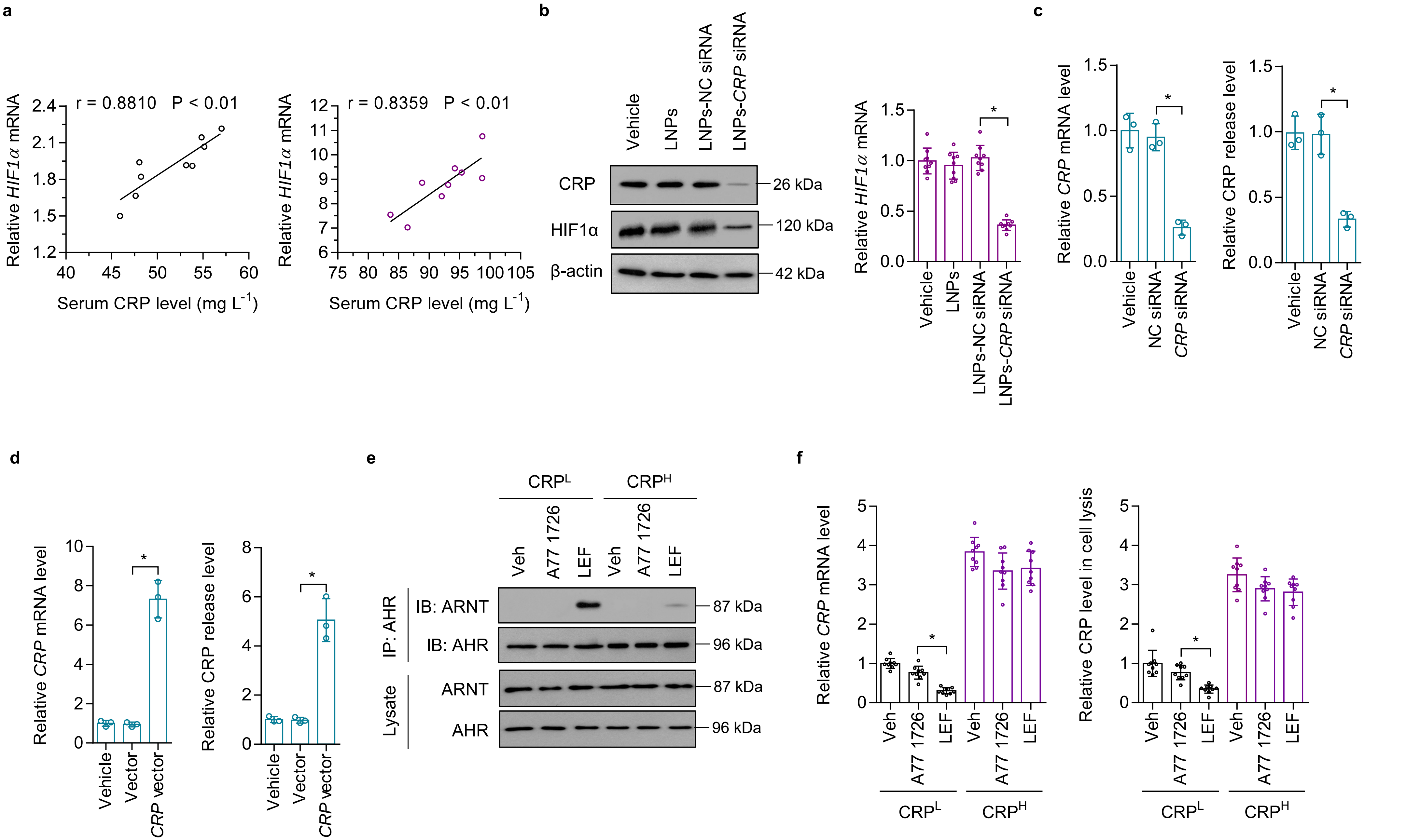
a
r = 0.8810 P < 0.01
Relative HIF1α mRNA
Serum CRP level (mg L-1)
r = 0.8359 P < 0.01
b
Vehicle
LNPs
LNPs-NC siRNA
LNPs-CRP siRNA
CRP
HIF1α
β-actin
26 kDa
120 kDa
42 kDa
c
Relative CRP mRNA level
Relative CRP release level
NC siRNA
CRP siRNA
d
Vector
CRP vector
e
CRPL
CRPH
Veh
A77 1726
LEF
IP: AHR
IB: ARNT
IB: AHR
Lysate
ARNT
AHR
87 kDa
96 kDa
f
Relative CRP level in cell lysis

Supplementary Fig. 8. Positive association between CRP and HIF1α *in vivo*. (**a**) Association between CRP level and relative *HIF1α* mRNA expression in CRP^L (left) and CRP^H (right) CIA rats. (**b**) Levels of CRP and HIF1α in hepatocytes from CRP^H CIA rats treated with PBS (Vehicle), LNPs, LNPs encapsulating negative control siRNA (LNPs-NC siRNA) or LNPs encapsulating CRP siRNA (LNPs-*CRP* siRNA), with a dosage of siRNA at 5.0 mg kg^{-1}. n = 9 for each group. (**c**) Relative levels of *CRP* mRNA and CRP release in human normal hepatocytes (THLE-2 cells) transfected with vehicle, *CRP* siRNA and negative control siRNA (NC siRNA), respectively. (**d**) Relative level of *CRP* mRNA and CRP release in THLE-2 cells transfected with vehicle, empty vector and *CRP* overexpressing vector, respectively. Each experiment was repeated three times. (**e**) Level of ARNT binding with HIF1α or AHR in hepatocytes from CRP^L and CRP^H CIA rats treated with Leflunomide, A77 1726 or vehicle. (**f**) Relative *CRP* mRNA (left) and CRP protein (right) levels in hepatocytes from the above rats. n = 9 for each group. * $P < 0.05$ as determined by one-way ANOVA with a *post-hoc* test. Source data are provided as a Source Data file.

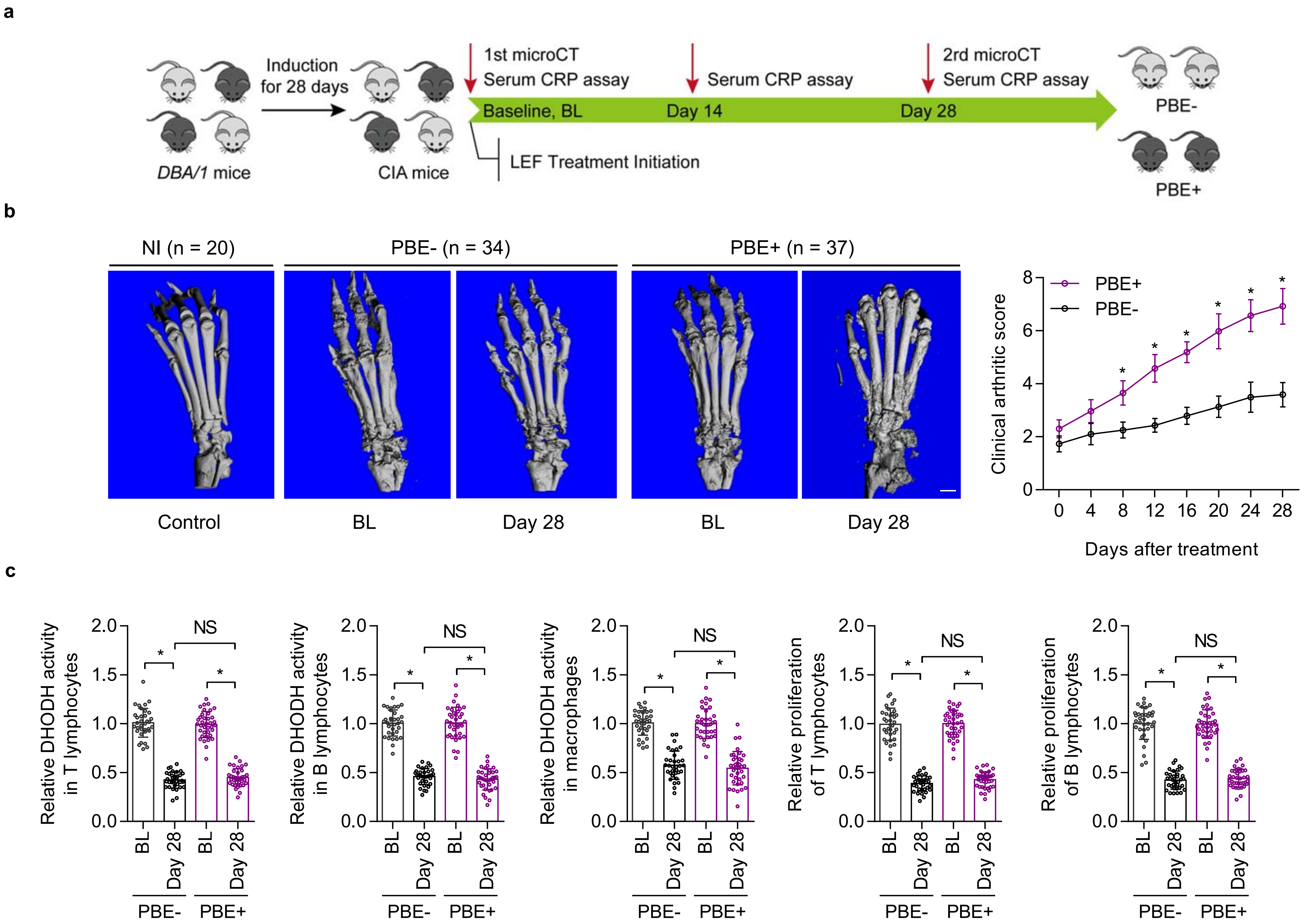

a
Induction for 28 days
DBA/1 mice
CIA mice
1st microCT
Serum CRP assay
Serum CRP assay
2rd microCT
Serum CRP assay
Baseline, BL
Day 14
Day 28
LEF Treatment Initiation
PBE-
PBE+
b
NI (n = 20)
PBE- (n = 34)
PBE+ (n = 37)
Control
BL
Day 28
BL
Day 28
PBE+
PBE-
Clinical arthritic score
Days after treatment
c
Relative DHODH activity in T lymphocytes
Relative DHODH activity in B lymphocytes
Relative DHODH activity in macrophages
Relative proliferation of T lymphocytes
Relative proliferation of B lymphocytes
NS
BL
Day 28
PBE-
PBE+

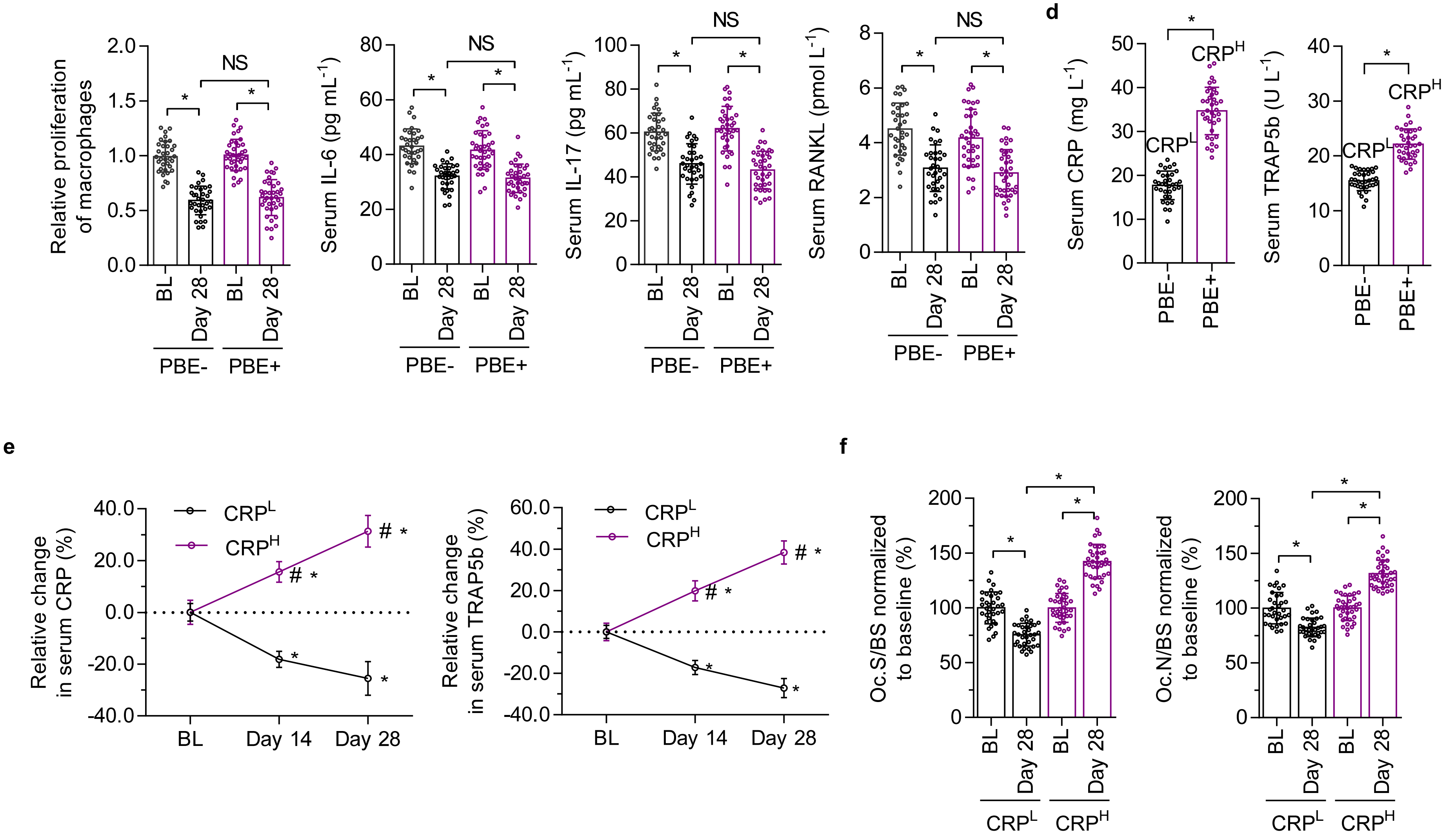
Relative proliferation of macrophages
2.0
1.5
1.0
0.5
0.0
NS
*
*
BL
Day 28
BL
Day 28
PBE-
PBE+
Serum IL-6 (pg mL-1)
80
60
40
20
0
Serum IL-17 (pg mL-1)
100
80
60
40
20
0
Serum RANKL (pmol L-1)
8
6
4
2
0
d
Serum CRP (mg L-1)
50
40
30
20
10
0
CRPL
CRPH
PBE-
PBE+
Serum TRAP5b (U L-1)
40
30
20
10
0
e
Relative change in serum CRP (%)
40.0
20.0
0.0
-20.0
-40.0
CRPL
CRPH
*
BL
Day 14
Day 28
Relative change in serum TRAP5b (%)
60.0
40.0
20.0
0.0
-20.0
-40.0
f
Oc.S/BS normalized to baseline (%)
200
150
100
50
0
CRPL
CRPH
Oc.N/BS normalized to baseline (%)

Supplementary Fig. 9. Differential responsiveness to Leflunomide among CIA mice. (**a**) The diagram of the experimental design. Briefly, *DBA/1* mice were immunized with bovine type II collagen for 28 days to establish the CIA models. The CIA mice were orally administered with Leflunomide for 28 days at a dose of 10.0 mg kg^{-1} d^{-1}. *In vivo* microCT analysis and serum CRP assay was performed before (baseline, BL) and after the treatment. (**b**) The representative three-dimensional microCT images and clinical arthritic score of the PBE+ (n = 37) and PBE- (n = 34) CIA mice. Non-immunized (NI) mice (n = 20) were used as the controls of the CIA mice. Scale bar, 1.0 mm. * $P < 0.05$ as determined by repeated measures ANOVA with a *post-hoc* test. (**c**) Relative levels of DHODH activity and proliferation of immune cells (T and B lymphocytes and macrophages) in synovial fluid and IL-6, IL-17 and RANKL levels in serum from PBE+ (n = 37) and PBE- (n = 34) CIA mice. The level of DHODH activity and proliferation of lymphocytes and macrophages were normalized to the corresponding baseline. * $P < 0.05$ as determined by one-way ANOVA with a *post-hoc* test, NS: no significance. (**d**) The serum baseline CRP and TRAP5b levels in PBE- (n = 34) and PBE+ (n = 37) CIA mice. CRP^L: relatively lower serum CRP level (17.70 ± 3.25 mg L^{-1}). CRP^H: higher serum CRP level (34.67 ± 5.41 mg L^{-1}). * $P < 0.05$ as determined by two-sided *t*-test. (**e**) Relative changes of serum CRP and TRAP5b from the corresponding baseline in CRP^H (n = 37) and CRP^L (n = 34) CIA mice. * $P < 0.05$ for day 14 or day 28 versus BL in CRP^L and CRP^H, # $P < 0.05$ for CRP^H versus CRP^L at day 14 and day 28, as determined by repeated measures ANOVA with a *post-hoc* test. (**f**) Bone resorption parameters including Oc.S/BS and Oc.N/BS after normalization to the corresponding baseline. * $P < 0.05$ as determined by one-way ANOVA with a *post-hoc* test. Source data are provided as a Source Data file.

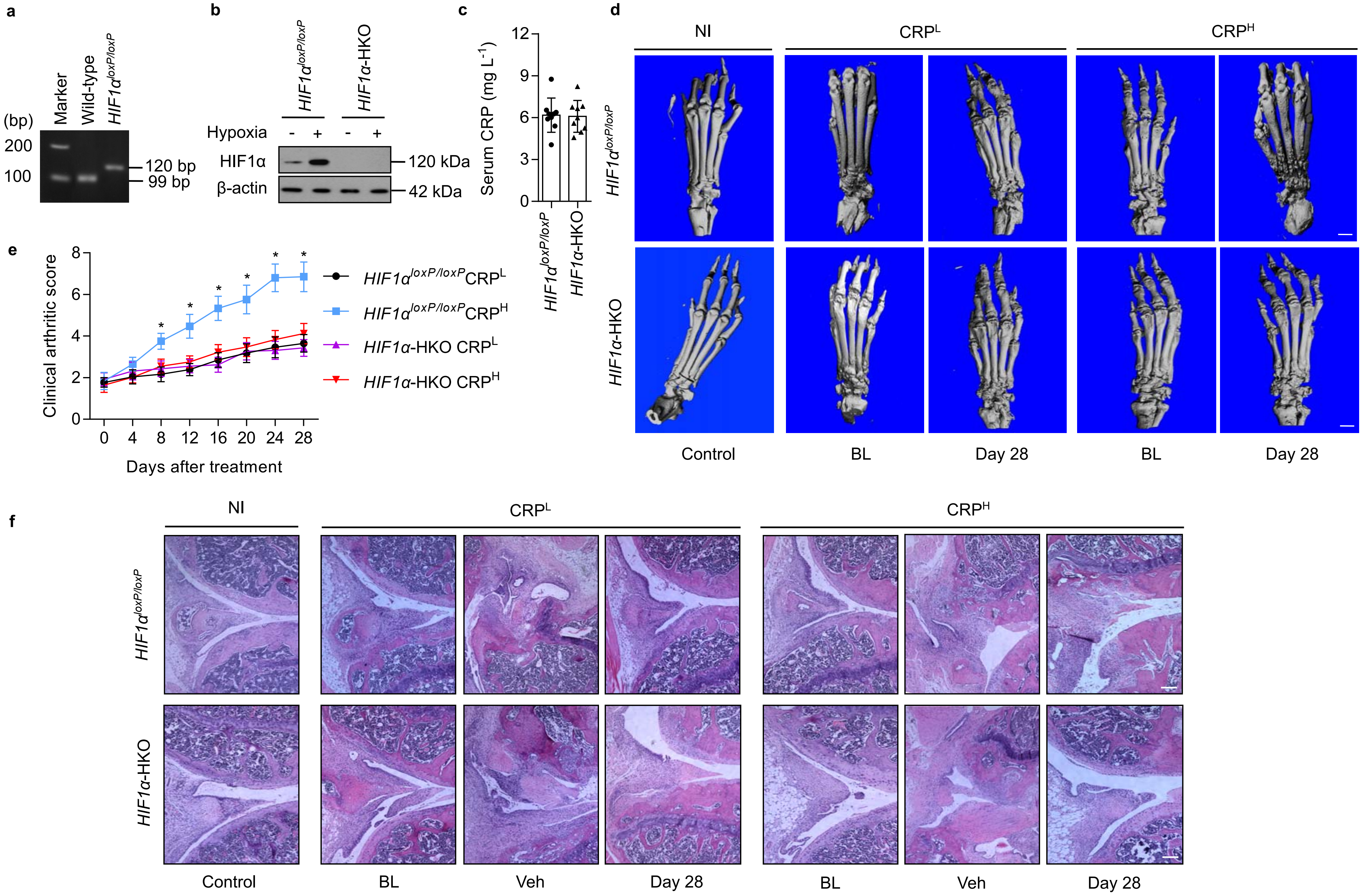

a
(bp)
Marker
Wild-type
HIF1α loxP/loxP
200
100
120 bp
99 bp
b
HIF1α loxP/loxP
HIF1α-HKO
Hypoxia
HIF1α
β-actin
120 kDa
42 kDa
c
Serum CRP (mg L-1)
d
NI
CRP L
CRP H
Control
BL
Day 28
e
Clinical arthritic score
Days after treatment
HIF1α loxP/loxP CRP L
HIF1α loxP/loxP CRP H
HIF1α-HKO CRP L
HIF1α-HKO CRP H
f
Veh

g

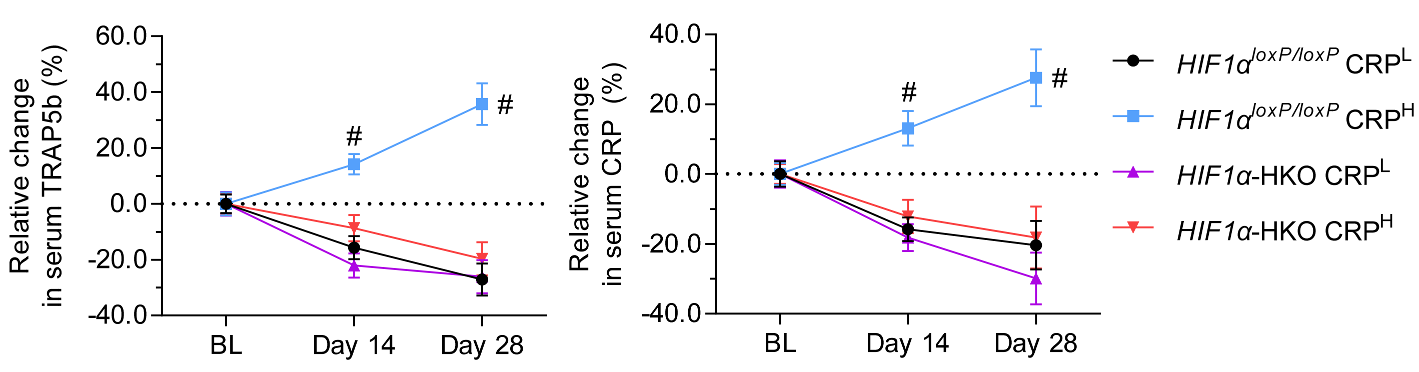
Relative change in serum TRAP5b (%)
60.0
40.0
20.0
0.0
-20.0
-40.0
BL
Day 14
Day 28
#
#
Relative change in serum CRP (%)
40.0
20.0
0.0
-20.0
-40.0
BL
Day 14
Day 28
#
#
HIF1α loxP/loxP CRP L
HIF1α loxP/loxP CRP H
HIF1α-HKO CRP L
HIF1α-HKO CRP H

h

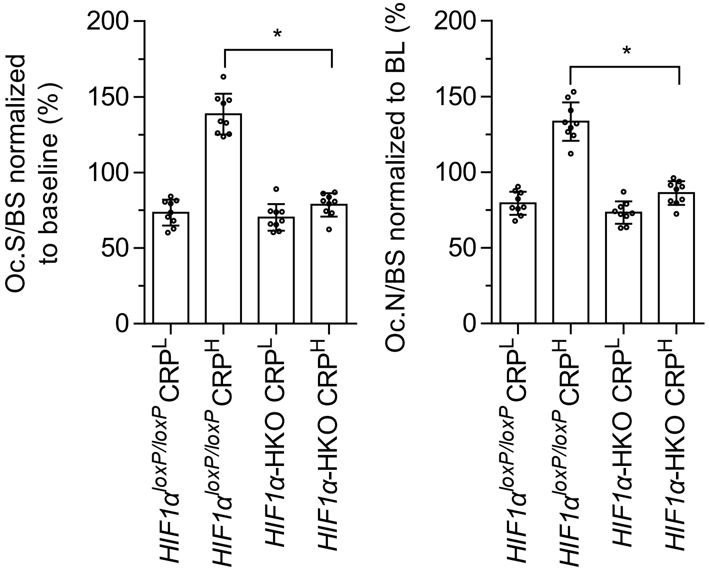
Oc.S/BS normalized to baseline (%)
200
150
100
50
0
*
HIF1α loxP/loxP CRP L
HIF1α loxP/loxP CRP H
HIF1α-HKO CRP L
HIF1α-HKO CRP H
Oc.N/BS normalized to BL (%)
200
150
100
50
0
*
HIF1α loxP/loxP CRP L
HIF1α loxP/loxP CRP H
HIF1α-HKO CRP L
HIF1α-HKO CRP H

i

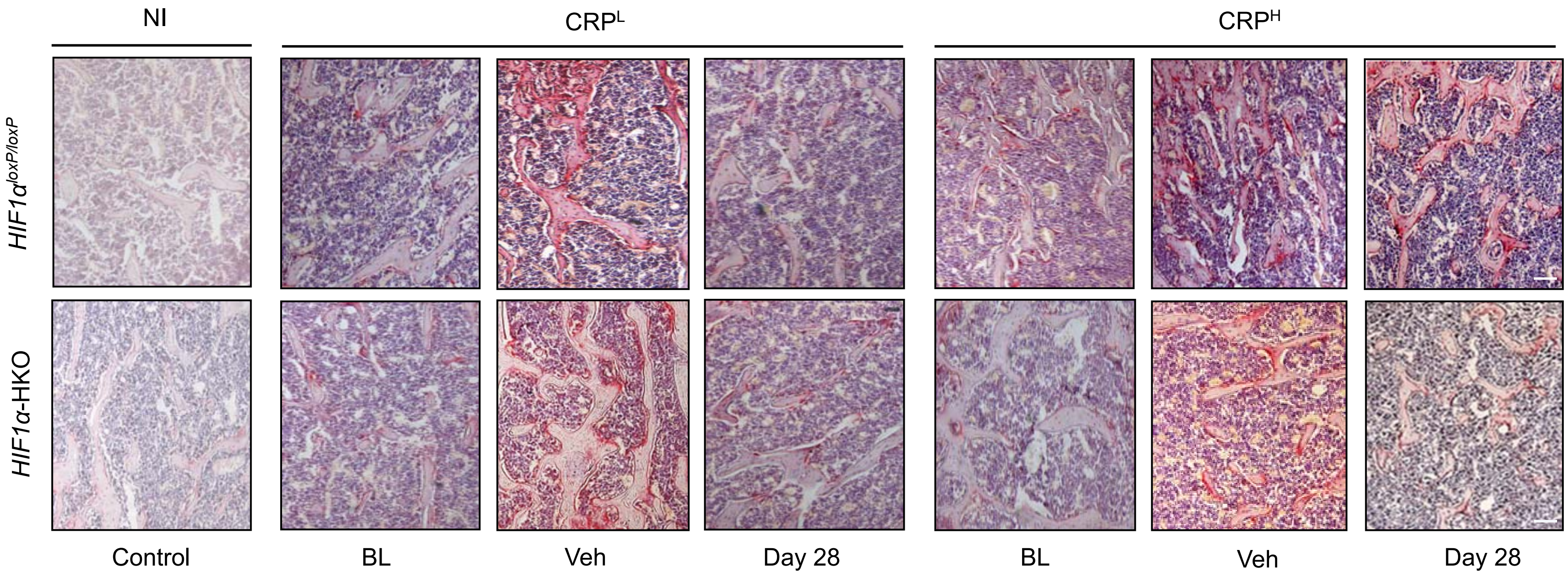
NI
CRP L
CRP H
HIF1α loxP/loxP
HIF1α-HKO
Control
BL
Veh
Day 28
BL
Veh
Day 28

Supplementary Fig. 10. Hepatocyte-specific deletion of *HIF1α* rescued the potency of Leflunomide in CRP^{H} CIA mice. (**a**) PCR analysis of the recombination of the *HIF1α floxed* allele. (**b**) HIF1α expression in hepatocytes from hepatocyte-specific *HIF1α* knockout (HIF1α-HKO) mice and control *HIF1α*$^{loxP/loxP}$ mice. Hepatocytes incubated in hypoxia were used as positive controls. (**c**) The serum CRP levels in *HIF1α*-HKO mice and control *HIF1α*$^{loxP/loxP}$ mice. * $P < 0.05$ as determined by two-sided *t*-test. (**d**) The representative three-dimensional micro-CT images of the CRP^{L} and CRP^{H} subgroups of *HIF1α*-HKO CIA mice and control *HIF1α*$^{loxP/loxP}$ CIA mice before (baseline, BL) and after the treatment. Scale bar, 1.0 mm. (**e**) Clinical arthritic scores of the CIA mice. * $P < 0.05$ as determined by repeated measures ANOVA with a *post-hoc* test. (**f**) Histological analysis by hematoxylin and eosin (H&E) staining to document synovial hyperplasia and inflammation in the CIA mice. Scale bar, 10.0 μm. (**g**) Changes of the serum CRP and TRAP5b from the corresponding baseline in the CIA mice. # $P < 0.05$ for CRP^{H} versus CRP^{L} at day 14 and day 28. # $P < 0.05$ for *HIF1α*$^{loxP/loxP}$ CRP^{H} versus *HIF1α*$^{loxP/loxP}$ CRP^{L} at day 14 and day 28 * $P < 0.05$ as determined by repeated measures ANOVA with a *post-hoc* test. (**h**) Bone resorption parameters including Oc.S/BS and Oc.N/BS normalized to the corresponding baseline. * $P < 0.05$ as determined by one-way ANOVA with a *post-hoc* test. (**i**) TRAP staining to document osteoclastic activity. Scale bar, 10.0 μm. n = 9 for each group. Source data are provided as a Source Data file.

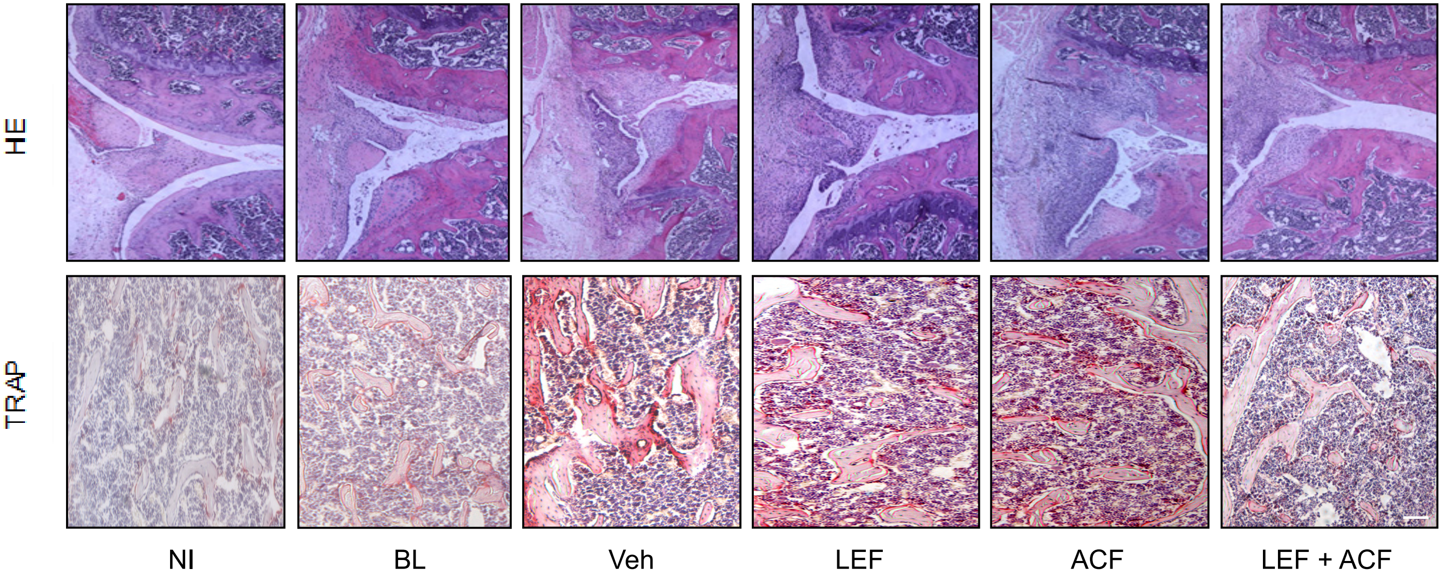
HE
TRAP
NI
BL
Veh
LEF
ACF
LEF + ACF

Supplementary Fig. 11. Inflammatory synovial hyperplasia and bone resorption in CRP^{H} CIA rats. Histological analysis by hematoxylin and eosin (H&E) staining to document inflammatory synovial hyperplasia in CRP^{H} CIA rats administered with vehicle (Veh), Leflunomide (LEF, 10.0 mg kg^{-1} d^{-1}), ACF (1.0 mg kg^{-1} d^{-1}) and the combination of Leflunomide and ACF (LEF + ACF, LEF, 10,0 mg kg^{-1} d^{-1}; ACF, 1.0 mg kg^{-1} d^{-1}) for 28 days, respectively. TRAP staining to document bone resorption in the CRP^{H} CIA rats. Scale bar, 40 μm.

Supplementary Table 1. The characteristics of RA patients before treatment

Characteristic	PBE- group	PBE+ group
Female/male	77/53	73/47
Age (years)	52 ± 11	53 ± 10
Duration (months)	12 ± 6	12 ± 8
RF positive (%)	78%	83%
Tender joint count	4.0 ± 2.1	4.9 ± 2.5
Swollen joint count	2.1 ± 1.2	2.8 ± 1.4
ESR (mm/h)	35.38 ± 6.53	41.09 ± 7.18
VAS pain (0-10)	2.2 ± 1.5	2.6 ± 1.2
DAS28-ESR	3.98 ± 0.59	4.24 ± 0.57
CRP (mg/l)	21.24 ± 8.94	51.17 ± 10.60

Supplementary Table 2. Indication accuracy of PBE+ RA patients using blood indicators

**CRP**					
Observed			Predicted		
			Group		Percentage Correct
			PBE-	PBE+	
	Group	PBE-	121	9	93.1
		PBE+	10	110	91.7
	Overall Percentage				92.4
**IGA**					
Observed			Predicted		
			Group		Percentage Correct
			PBE-	PBE+	
	Group	PBE-	86	44	66.2
		PBE+	45	75	62.5
	Overall Percentage				64.4
**IGG**					
Observed			Predicted		
			Group		Percentage Correct
			PBE-	PBE+	
	Group	PBE-	99	31	76.2
		PBE+	90	30	25.0
	Overall Percentage				51.6
**IGM**					
Observed			Predicted		
			Group		Percentage Correct
			PBE-	PBE+	
	Group	PBE-	98	32	75.4
		PBE+	86	34	28.3
	Overall Percentage				52.8
**ESR**					
Observed			Predicted		
			Group		Percentage Correct
			PBE-	PBE+	
	Group	PBE-	93	37	71.5
		PBE+	69	51	42.5
	Overall Percentage				57.6
**Anti-CCP**					
Observed			Predicted		
			Group		Percentage Correct
			PBE-	PBE+	
	Group	PBE-	90	40	69.2
		PBE+	55	65	54.2
	Overall Percentage				62.0

Supplementary Table 3. Tissue distribution of LNPs-*CRP* siRNA

Organ	Percent of dose	
	1.0 h	3.0 h
Brain	0.01 ± 0.00	0.01 ± 0.00
Heart	0.13 ± 0.04	0.05 ± 0.02
Kidneys	0.42 ± 0.08	0.25 ± 0.04
Liver	90.35 ± 6.36	88.86 ± 8.79
Lungs	0.47 ± 0.06	0.30 ± 0.04
Pancreas	0.07 ± 0.02	0.03 ± 0.01
Spleen	4.84 ± 0.85	4.53 ± 0.76
Stomach	0.10 ± 0.04	0.04 ± 0.01

Supplementary Table 4. Theoretical parameters of Leflunomide-AHR interaction

Mode	Affinity (kcal/mol)	Dist from best mode		Residues
		rmsd l.b.	rmsd u.b.	
1	-8.0	0.000	0.000	H291, K303
2	-7.8	1.234	2.212	H291, V381
3	-7.5	1.023	2.733	H291, V381, K303, F324
4	-6.7	5.035	7.492	F324, K303
5	-5.9	4.997	6.814	M348
6	-5.6	4.984	7.101	S365, S346

Supplementary Table 5. Biochemistry and hematology parameters in CRP^H CIA rats

Group	ALT (U L^{-1})	AST (U L^{-1})	TP (g dL^{-1})	BUN (mg dL^{-1})	RBC (x10^6 μL^{-1})	Hemoglobin (g dL^{-1})	WBC (x10^3 μL^{-1})	PLT (x10^3 μL^{-1})
BL	25.2 ± 2.6	76.4 ± 5.4	5.3 ± 0.7	22.2 ± 2.9	9.2 ± 1.6	16.2 ± 1.3	2.9 ± 0.5	974.4 ± 110.1
Veh	27.5 ± 3.5	79.5 ± 8.2	5.4 ± 0.5	25.1 ± 3.3	8.9 ± 2.2	16.0 ± 2.8	3.1 ± 0.4	988.5 ± 115.2
LEF	28.2 ± 4.3	82.4 ± 9.8	5.2 ± 0.6	25.4 ± 4.1	8.8 ± 1.2	15.8 ± 3.3	3.3 ± 0.5	995.3± 120.8
ACF	28.6 ± 3.9	83.2 ± 8.5	5.4 ± 0.7	23.1 ± 4.7	8.4 ± 0.9	15.9 ± 1.6	3.1 ± 0.6	990.1 ± 130.7
LEF + ACF	31.4 ± 4.1	81.7 ± 7.9	5.5 ± 0.6	25.7 ± 3.9	8.5 ± 1.8	15.5 ± 2.5	3.0 ± 0.4	980.7 ± 110.4

ALT, Alanine aminotransferase; AST, aspartate aminotransferase; BUN, blood urea nitrogen; TP, total protein; WBC, white blood cell; RBC, red blood cell; PLT, platelet; BL, baseline; Veh, vehicle; LEF, Leflunomide; ACF, Acriflavine; LEF + ACF, the combination of Leflunomide and Acriflavine.
