# Supplementary material for: HIF1α inhibition facilitates Leflunomide-AHR-CRP signaling to attenuate bone erosion in CRP-aberrant rheumatoid arthritis
Source: Nat Commun. 2019 Oct 8;10:4579. doi: 10.1038/s41467-019-12163-z (PMC6783548; doi:10.1038/s41467-019-12163-z)
Supplement: Supplementary file 3 — Reporting Summary [file 41467_2019_12163_MOESM3_ESM.pdf]

nature research

Corresponding author(s): Aiping Lu, Ge Zhang, Bao-Ting Zhang, Chao Liang

Last updated by author(s): Jul 18, 2019

Reporting Summary

Nature Research wishes to improve the reproducibility of the work that we publish. This form provides structure for consistency and transparency in reporting. For further information on Nature Research policies, see Authors & Referees and the Editorial Policy Checklist.

Statistics

For all statistical analyses, confirm that the following items are present in the figure legend, table legend, main text, or Methods section.

n/a	Confirmed	
☐	☒	The exact sample size (n) for each experimental group/condition, given as a discrete number and unit of measurement
☐	☒	A statement on whether measurements were taken from distinct samples or whether the same sample was measured repeatedly
☐	☒	The statistical test(s) used AND whether they are one- or two-sided<br>*Only common tests should be described solely by name; describe more complex techniques in the Methods section.*
☐	☒	A description of all covariates tested
☐	☒	A description of any assumptions or corrections, such as tests of normality and adjustment for multiple comparisons
☐	☒	A full description of the statistical parameters including central tendency (e.g. means) or other basic estimates (e.g. regression coefficient) AND variation (e.g. standard deviation) or associated estimates of uncertainty (e.g. confidence intervals)
☐	☒	For null hypothesis testing, the test statistic (e.g. F, t, r) with confidence intervals, effect sizes, degrees of freedom and P value noted<br>*Give P values as exact values whenever suitable.*
☒	☐	For Bayesian analysis, information on the choice of priors and Markov chain Monte Carlo settings
☒	☐	For hierarchical and complex designs, identification of the appropriate level for tests and full reporting of outcomes
☐	☒	Estimates of effect sizes (e.g. Cohen's d, Pearson's r), indicating how they were calculated

Our web collection on statistics for biologists contains articles on many of the points above.

Software and code

Policy information about availability of computer code

Data collection	No software was used.
Data analysis	No software was used.

For manuscripts utilizing custom algorithms or software that are central to the research but not yet described in published literature, software must be made available to editors/reviewers. We strongly encourage code deposition in a community repository (e.g. GitHub). See the Nature Research guidelines for submitting code & software for further information.

Data

Policy information about availability of data

All manuscripts must include a data availability statement. This statement should provide the following information, where applicable:

- Accession codes, unique identifiers, or web links for publicly available datasets
- A list of figures that have associated raw data
- A description of any restrictions on data availability

The authors declare that all data supporting the findings of this study are available within the article and its Supplementary Information files or are available from the authors upon reasonable request. The source data underlying Fig. 1-6, Supplementary Fig. 1-4 and Supplementary Fig. 6-10 are provided as a Source Data file.

Field-specific reporting

Please select the one below that is the best fit for your research. If you are not sure, read the appropriate sections before making your selection.

☒ Life sciences ☐ Behavioural & social sciences ☐ Ecological, evolutionary & environmental sciences

Life sciences study design

All studies must disclose on these points even when the disclosure is negative.

Sample size	Sample size was calculated by the formula: n = 2 [(U?+Uß) S δ-1]2. S in the formula referred to the standard deviation (s.d.). δ in the formula represented the inter-group difference of the mean values. There was a difference above 30 mg cm-3 in BMD between treatment group and control group, i.e., δ = 30 mg cm-3. s.d. of BMD from previous studies was 15 mg cm-3, i.e., S = 15 mg cm-3. We selected the significant level at 5% (? = 0.05) in a two-tailed test and power of the study at 90% (1- ß = 0.9). According to the formula: n = 2 [(U?+Uß) S δ-1]2, U0.05 = 1.960, ß = 0.10, U0.1 = 1.282, we chose n = 9 for each group, which was enough to detect the real difference among the treatment groups. Please refer to "Statistical analysis" in Materials and Methods.
Data exclusions	Animals in poor body condition, such as tumors or other sick conditions during aging, were excluded. The exclusion was made before random group assignments, experimental intervention and data analysis. Please refer to "Statistical analysis" in Materials and Methods.
Replication	Two types of replicates including biological replicates and technical replicates was performed to yield accurate and reliable statistics. All attempts at replication were successful. Please refer to "Statistical analysis" in Materials and Methods.
Randomization	Samples were randomly located into different groups. Please refer to "Statistical analysis" in Materials and Methods.
Blinding	The investigators were blinder to group allocation during data collection and analysis. Please refer to "Statistical analysis" in Materials and Methods.

Reporting for specific materials, systems and methods

We require information from authors about some types of materials, experimental systems and methods used in many studies. Here, indicate whether each material, system or method listed is relevant to your study. If you are not sure if a list item applies to your research, read the appropriate section before selecting a response.

Materials & experimental systems

n/a	Involved in the study	
☐	☒	Antibodies
☐	☒	Eukaryotic cell lines
☒	☐	Palaeontology
☐	☒	Animals and other organisms
☒	☐	Human research participants
☒	☐	Clinical data

Methods

n/a	Involved in the study	
☒	☐	ChIP-seq
☒	☐	Flow cytometry
☒	☐	MRI-based neuroimaging

Antibodies

Antibodies used	Antibodies included an anti-AHR antibody (ab2769, 1: 500), an anti-ARNT antibody (ab5624, 1: 500), an anti-HIF1α (ab1, 1: 200) antibody, an anti-CRP antibody (ab50861, 1: 2000), an anti-VEGFA antibody (ab46154, 1: 1000), an anti-JunD antibody (ab28837, 1: 500), an anti-cyclin A antibody (ab181591, 1: 2000), an anti-Src antibody (ab47405, 1: 500), an anti-Src (phospho Y418) antibody (ab4816, 1: 1000) and an anti-β-actin (ab8226, 1: 10000) antibody from Abcam.
Validation	Antibodies were validated for the specific species and application in pilot studies. Please refer to "Immunoprecipitation and western blots" in Materials and Methods.

Eukaryotic cell lines

Policy information about cell lines

Cell line source(s)	Human normal hepatocyte cell line THLE-2 (CRL-2706) was from ATCC. Please refer to "Cell culture" in Materials and Methods.
Authentication	Different cells used for in vitro studies was authenticated by flow cytometry. Please refer to Materials and Methods.
Mycoplasma contamination	No mycoplasma contamination was confirmed for the cell line. Please refer to "Cell culture" in Materials and Methods.
Commonly misidentified lines (See ICLAC register)	There was no commonly misidentified cell line used in this study. Please refer to "Cell culture" in Materials and Methods.

Animals and other organisms

Policy information about studies involving animals; ARRIVE guidelines recommended for reporting animal research

Laboratory animals	Male Sprague-Dawley (SD) rats (170 g-190 g of weight) were from the Chinese University of Hong Kong and kept with free access to food and water under standard temperature conditions (22°C) and a 12h light/dark cycle. Male DBA/1 mouse aged 8-10 weeks were from the Chinese University of Hong Kong and kept with free access to food and water under standard temperature conditions (22°C) and a 12h light/dark cycle. Please refer to "CIA rat model" and "CIA mouse model" in Materials and Methods.
Wild animals	No wild animals were used in this study.
Field-collected samples	No wild animals were used in this study.
Ethics oversight	All the experimental procedures were approved by the Committees of Animal Ethics and Experimental Safety of the Hong Kong Baptist University. Please refer to "CIA rat model" and "CIA mouse model" in Materials and Methods.

Note that full information on the approval of the study protocol must also be provided in the manuscript.
